# Self-excited valve using a flat ring tube: Application to robotics

**DOI:** 10.3389/frobt.2022.1008559

**Published:** 2022-11-29

**Authors:** Hiroyuki Nabae, Eigo Kitamura

**Affiliations:** Department of Mechanical Engineering, Tokyo Institute of Technology, Tokyo, Japan

**Keywords:** self-excitation, pneumatic actuator, soft robot, soft valve, locomotive robot

## Abstract

Complex and bulky driving systems are among the main issues for soft robots driven by pneumatic actuators. Self-excited oscillation is a promising approach for dealing with this problem: oscillatory actuation is generated from non-oscillatory input. However, small varieties of self-excited pneumatic actuators currently limit their applications. We present a simple, self-excited pneumatic valve that uses a flat ring tube (FRT), a device originally developed as a self-excited pneumatic actuator. First, we explore the driving principle of the self-excited valve and investigate the effect of the flow rate and FRT length on its driving frequency. Then, a locomotive robot containing the valve is demonstrated. The prototype succeeded in walking at 5.2 mm/s when the oscillation frequency of the valve was 1.5 Hz, showing the applicability of the proposed valve to soft robotics.

## 1 Introduction

Soft robots exhibit various interesting characteristics owing to their soft/flexible bodies, enabling them to perform a range of tasks, including the handling of fragile and/or complex shaped objects without sensors and locomotion with shape adaptability to a complex and narrow environment like inside pipelines. The flexible motions of soft robots are achieved with various types of actuators (El-Atab et al., 2020), including ionic-polymer–metal composites (Oguro et al., 1993; Bhandari et al., 2012) and dielectric elastomer actuators (O’Halloran et al., 2008). Soft actuators driven by pneumatic/hydraulic pressure (Suzumori et al., 1991; Onal and Rus 2012; Rus and Tolley 2015; Rajappan et al., 2021) are particularly simple in terms of structure and operating principle, with the further advantages of a high output-to-weight ratio and large deformation.

However, the size and complexity of fluidic actuators, especially multi-degree-of-freedom drive systems, pose major challenges. Conventional pneumatic-drive systems mainly consist of pumps for the pressure source, valves to control the applied pressure, and air hoses for transmitting the air pressure. Multiple pressure-supply tubes and solenoid valves are required to operate each actuator in a conventional pneumatic system; kinks and friction in the numerous tubes can interfere with the robot’s movement and motion, while the use of multiple solenoid valves reduces flexibility, adds weight, and complicates control.

Various solutions have been proposed for this problem [e.g., integratable/portable gas sources (Lloyd and Kim 2007; Shepherd et al., 2013; Wehner et al., 2016; Mirvakili et al., 2020) and electrically drivable pressure sources (Suzumori et al., 2013; Cacucciolo et al., 2019) (liquid type)]. This study focuses on using self-excited oscillation to simplify the drive system. Self-excited oscillation uses non-vibratory input to induce oscillating motion and can simplify pneumatic systems by unifying the drive system and actuator. Various methods of inducing self-excited oscillation have been used successfully in robots and mechatronic devices, including link mechanisms and electromagnetic motors (Nakashima et al., 2003; Cunningham and Asada 2009), chemical reaction (Maeda et al., 2007), electrostatics (Zhu et al., 2021), and thermal drives (Nemoto and Yamamoto 2015). Self-excited oscillation has also been utilized in pneumatic systems to simplify configuration and manufacturing; however, the motions and characteristics of existing self-excited pneumatic actuators are limited, and their applications are correspondingly restricted. Despite extensive recent research (Miyaki and Tsukagoshi 2018; Rothemund et al., 2018; Preston et al., 2019; Drotman et al., 2021; Hubbard et al., 2021), most pneumatic valves or circuits generating self-excited oscillation have complex structures with multiple components.

In the present study, we aim to realize a simple self-excited pneumatic valve that can switch between opening and closing of pressure by self-excited oscillation using only structural members. We focus on the flat ring tube (FRT) method (Tsukagoshi et al., 2007), in which self-excited motion is generated by simply bending a tube with a flat cross section. We design a valve incorporating an FRT; the applicability of the self-excited pneumatic valve to soft robotics is demonstrated with a prototype locomotive robot.

## 2 Characterization of self-excited valve using flat ring tube

### 2.1 Driving principle

**FIGURE 1:**
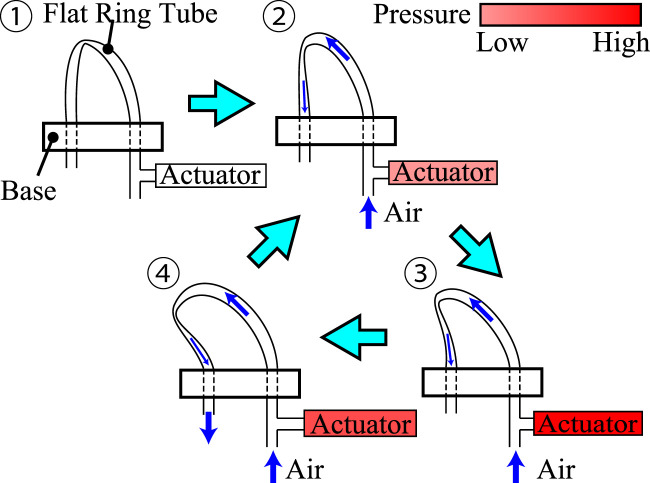
Self-excited oscillation of a flat ring tube produces cyclic pressure changes in an actuator.

The FRT was originally developed by Tsukagoshi et al. as an actuator (Tsukagoshi et al., 2007; Tsukagoshi et al., 2008). It is a thermoplastic tube flattened by heat processing. When the tube is curved and a fluid flows through it, a self-excited phenomenon is caused by the changes in fluid pressure and the movement of the tube’s buckling point. In the initial state, the buckling point is close to the inlet of the tube; it moves as the applied pressure is increased. When the buckling point reaches the end of the tube, air is discharged and the buckling point returns to its original position. The tube performs self-excited oscillation by repeating this process. In this study, we use an FRT not as an actuator itself but in a self-excited valve that uses the change in pressure at the inlet of the tube to drive an actuator. This is illustrated in Figure 1. Because of the inflow of air, the pressure at the inlet increases as the buckling point moves; when the system reaches state 4, the air is released and the pressure falls. Thus, the internal pressure changes as the tube oscillates, causing repetitive motion of the pneumatic actuators.

### 2.2 Manufacturing process for the prototypes

This subsection describes the manufacture of a valve using FRTs. The procedure used was as follows:

1. A urethane tube with an outer diameter of 8 mm and an inner diameter of 6 mm was clipped with a metal plate of a specified length and secured in a vise.
2. The tube was placed in a furnace and heated at 100°C for 1 hour.
3. After being removed from the furnace and allowed to cool, the tube was removed from the vise.

In addition, a metal plate was bound together with the urethane tubes to maintain a constant thickness when the urethane tube was flattened.

### 2.3 Experimental characterization

**FIGURE 2:**
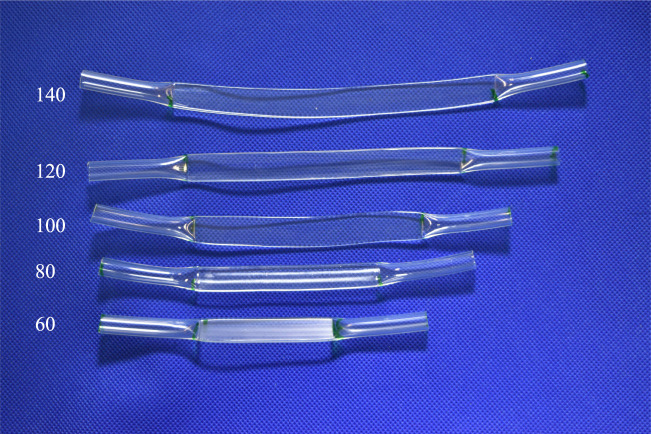
Manufactured flattened tubes.

**FIGURE 3:**
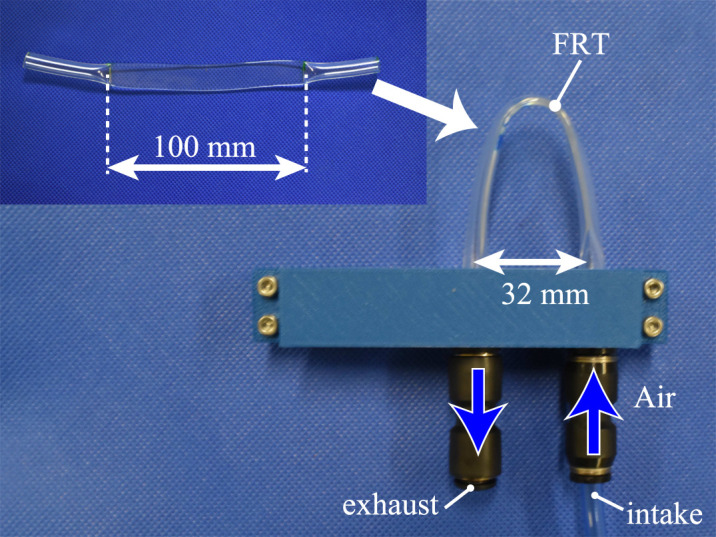
Valve with Flat Ring Tube (FRT) used in the experiment.

This subsection describes measurements confirming that the pressures generated by the proposed method are high enough to drive pneumatic actuators. The FRTs used in the experiment are shown in Figure 2; the prototype valves were of the form depicted in Figure 3.

**FIGURE 4:**
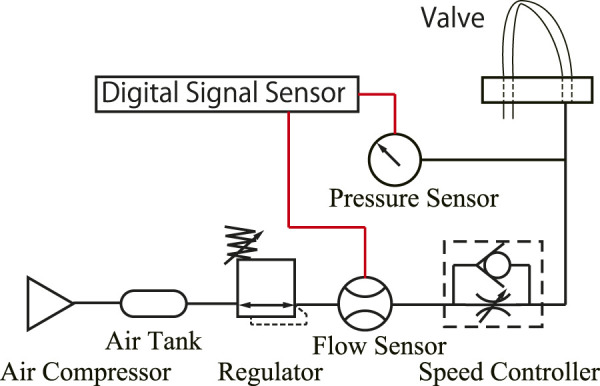
Setup for pressure measurement.

Here, we investigate the effect of the shape of the tube on vibration. The magnitude of pressure and vibration frequency were investigated by changing the inter-axial distance *w* [mm] between the air inlet and outlet of the FRT (32 mm in Figure 3) and the length *L* [mm] of the flattened tube (32 mm in Figure 3). The combinations investigated were 60, 80, 100, 120, and 140 mm for *L* and 8, 16, 24, 32, 40, 48, and 52 mm for *w*. Figure 4 shows the experimental setup. The inflow air pressure was regulated to 0.4 MPa. The flow rate was controlled by a speed controller. To minimize the effect of the volume of the air being pressurized, we configured the experimental setup such that only the air tube is between the speed controller and the valve. The flow rate and pressure were measured using a flowmeter (FD-A100, KEYENCE) and pressure gauge (AP-C33, KEYENCE), respectively; the results were recorded by a digital signal-processing system (DS1108, DSPACE).

**FIGURE 5:**
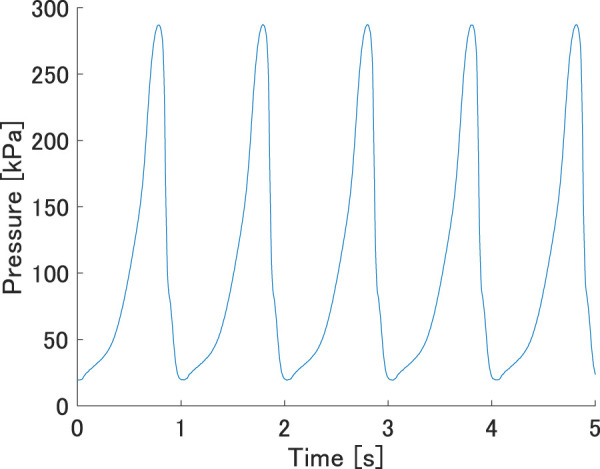
Typical waveform of the pressure measurement. The length, inter-axial width, and flow rate were 100 mm, 32 mm, and 15 L/min, respectively.

Figure 5 shows a typical waveform from a pressure measurement with *L* = 100 mm, *w* = 32 mm, and flow rate = 15 L/min. As shown in the figure, the prototype successfully generated oscillating pressure; the minimum and maximum pressures within one cycle were approximately 0.021 MPa and 0.287 MPa, respectively, a pressure differential sufficient to move many pneumatic actuators. The pressure was measured with a flow meter varying from 5 L/min to 250 L/min. If the vibration did not show a constant period or the frequency was less than 1/20 Hz, the system was not considered to be oscillating.

Table 1 summarizes the experimental results from the viewpoint of whether it oscillates or otherwise according to the parameters *L* and *w*. We discuss the effects of each parameter separately in the following sections.

**TABLE 1:** Experimental result. -:Not vibrating. •:Vibrating at some flow rates. ◦:Vibrating.

*L* [mm] *w* [mm]	8	16	24	32	40	48	52
60	-	-	-	•	-	-	-
80	-	-	-	○	•	•	-
100	-	-	•	•	-	-	-
120	-	-	•	○	○	•	-
140	-	-	-	○	○	○	-

**FIGURE 6:**
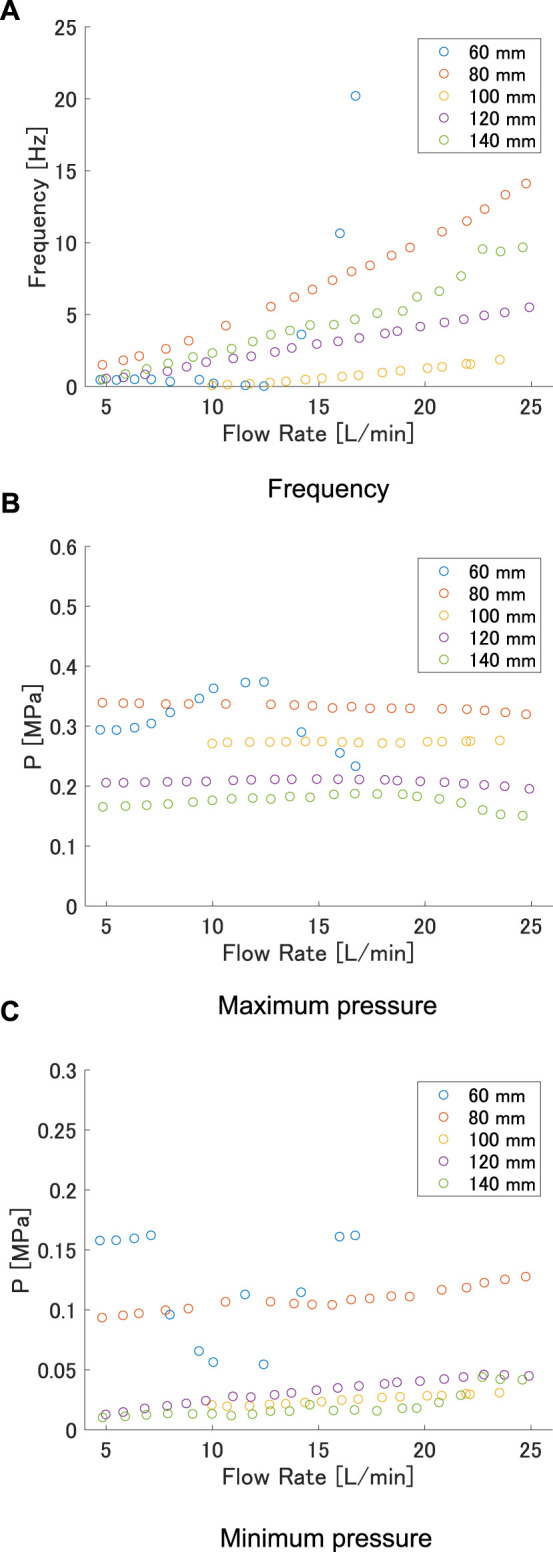
**(A)** Frequency, **(B)** maximum pressure, and **(C)** minimum pressure versus flow rate at each length when interaxial distance was 32 mm.

First, we discuss the effect of *L* for the case of *w* = 32 mm based on the relationship between flow rate and the measured values of frequency and pressure of maximum and minimum points. The relationship between flow rate and frequency is shown in Figure 6A, and that between flow rate and maximum and minimum pressures is shown in Figures 6B,C, respectively. As the flow rate oscillated, the average of the total flow rate was used for evaluation. For all lengths, the frequency generally increased with flow rate. This effect tended to decrease in the length range 60 mm–100 mm, but increased again from 100 mm to 140 mm. From Figure 6B and Figure 6C, the maximum and minimum pressures were not affected by the change in flow rate. However, they both decreased as *L* increased.

**FIGURE 7:**
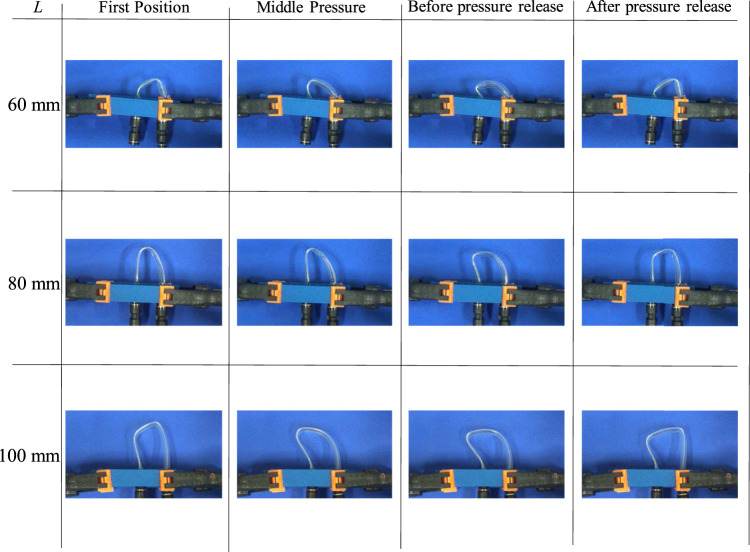
State of FRT before and after opening the valve.

The reason for the higher pressure at shorter lengths is the shape of the FRT near the buckling point during air release. Figure 7 shows the FRT before and after pressure release, i.e., at the highest and lowest pressures, for lengths of 60, 100, and 140 mm, respectively. It can be seen that the shorter FRTs had less curvature at the buckling point, making it more difficult for air to flow through them. Therefore, pressure release was more difficult, and the maximum pressure was higher. The inlet side of the FRT was also under high pressure after a pressure release of 60 mm, indicating that the FRT was bulging. This shows that the pressure release was difficult; even after it was released, the pressure was still high and closure would be expected to occur again. Thus, the maximum and minimum pressures were higher for shorter lengths. The trend of the oscillation frequency changed at *L* = 100 mm because of the aforementioned pressure release.

Figure 6C shows no significant change in minimum pressure with flowrate at 100 mm, 120 mm, or 140 mm. However, the maximum pressure decreased with increasing length. Therefore, the flow rate required for oscillation decreased as the length increased, and the oscillation frequency also decreased. From 60 mm to 100 mm, both the maximum and minimum pressures decreased, but the minimum pressure decrease was larger; this increased the flow rate required per vibration and decreased the vibration frequency.

**FIGURE 8:**
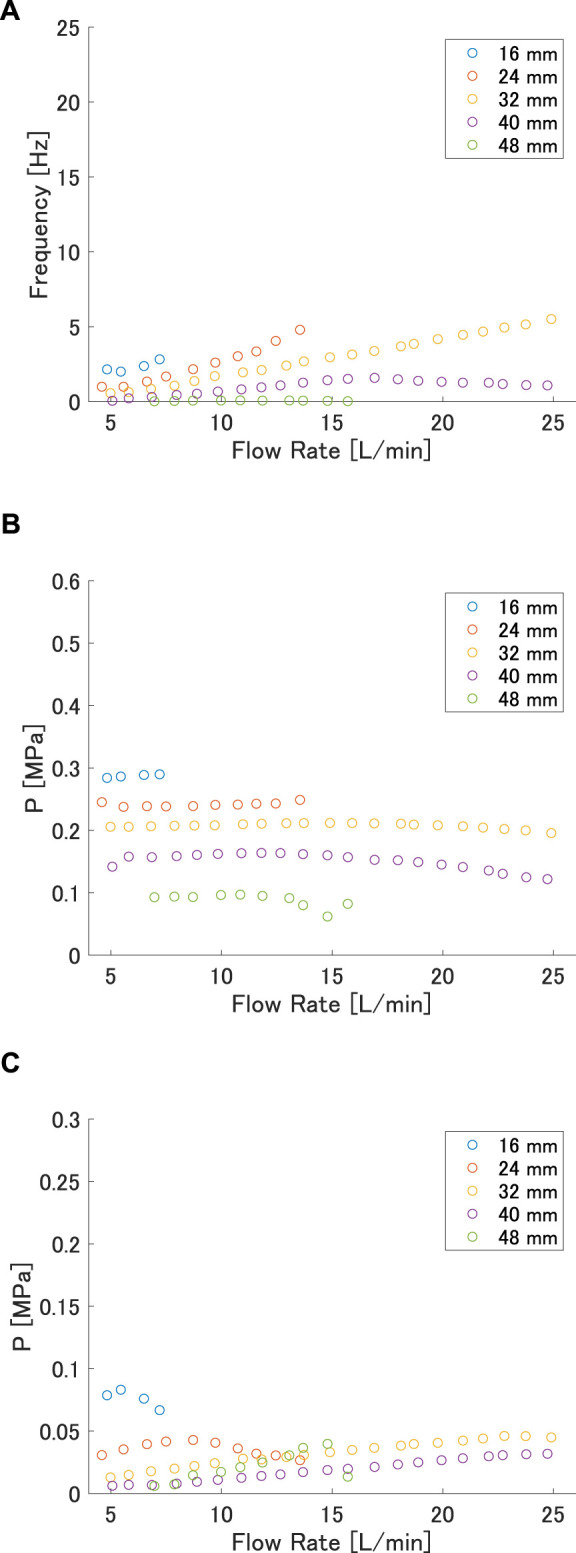
**(A)** Frequency, **(B)** maximum pressure, and **(C)** minimum pressure versus flow rate at each interaxial distance for length 120 mm.

The effect of *w* is also discussed for the case of *L* = 120 mm. The relationship between flow rate and frequency for *L* = 120 mm is shown in Figure 8A, and that between flow rate and maximum and minimum pressures is shown in Figure 8B and Figure 8C, respectively. From Figure 8A, it can be seen that the frequency, maximum pressure, and minimum pressure all decreased as the interaxial distance *w* increased. The maximum pressure decreased with increasing *w* in both Figure 8B and Figure 8C. The minimum pressure decreased as *w* increased, although the values became almost the same after 32 mm. The reason for the higher frequency and higher maximum pressure at shorter *w* is that the radius of curvature near the buckling point was smaller at smaller interaxial distances, just as it was at smaller lengths.

In summary:

• As the flow rate increased, the frequency increased, but the magnitude of the pressure was not affected.
• As the inter-axial distance and FRT length increased, both pressure and frequency decreased.

## 3 Application to locomotive soft robot

### 3.1 Design of locomotive robot

**FIGURE 9:**
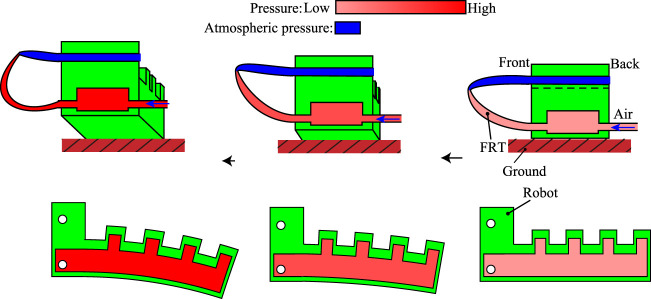
Principle of proposed robot: The legs begin to bend as the pressure of the FRT increases. The direction of bending is downward and backward, depending on the shape of the air chamber.

Consider a robot that walks by swinging its legs back and forth and up and down in a vibratory motion. A simple, easily manufactured actuator with a bellows structure is used, because such actuators are effective in situations where displacement in only one direction is required. The principle by which the robot walks is shown in Figure 9. The actuator is attached to the base of the FRT and moves in accordance with the latter’s vibration.

The entire walking robot is made of flexible material with holes where the FRTs are attached. The legs bend backward (allowing the robot to move forward) and downward (allowing only the legs to touch the ground) in response to increased pressure.

The robot has a bellows structure on each side, and was designed to bend and walk as the pressure in the bellows structure increased. The finite-element software ANSYS 2019R3 (ANSYS Inc.) was used to determine the pressure required to operate the robot. Large deformation was turned on in the analysis settings, and the nonlinear-mechanical mesh method (suitable for models with large nonlinearities such as flexible materials) was used. The material used was assumed to be the two-component room-temperature-vulcanized rubber KE-1600 (Shin-Etsu Chemical Co., Ltd.); the physical properties of KE-1600 are a density of 1.27 g/cm^3^, Durometer (Type A) 45, and tensile strength of 6.5 MPa. The third-order Ogden model was used to represent the hyperelastic properties necessary for the analysis, assuming that KE-1600 is completely incompressible. The strain energy potential *W* in this model is expressed as 1:

$$W=\overset{N}{\underset{i=1}{\sum }}\frac{\mu_{i}}{\alpha_{i}}\left({\bar{\lambda_{1}}}^{\alpha_{i}}+{\bar{\lambda_{2}}}^{\alpha_{i}}+{\bar{\lambda_{3}}}^{\alpha_{i}}-3\right),$$ (1)

where the material constants *λ*_*i*_ and *α*_*i*_ are calculated using the parameters in (Pramudita et al., 2017).

**FIGURE 10:**
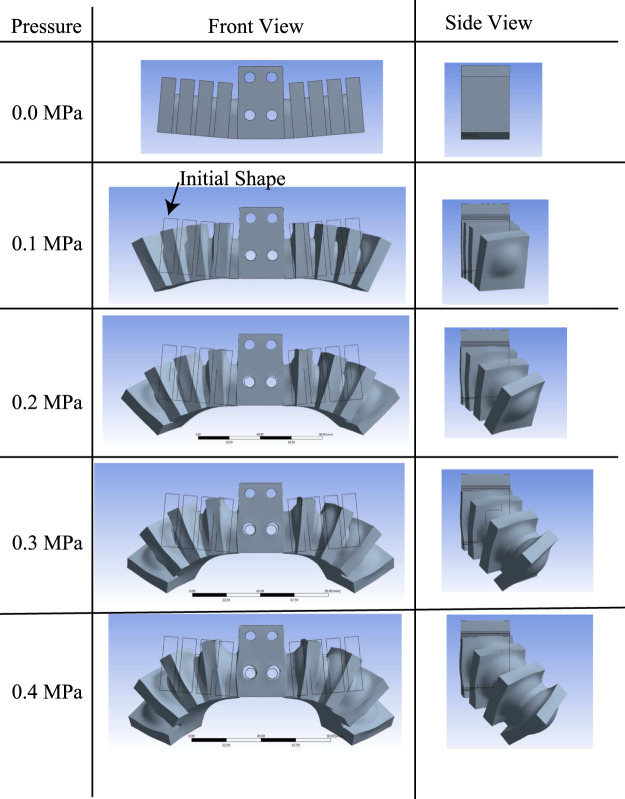
Analysis result by Finite-element-method.

**FIGURE 11:**
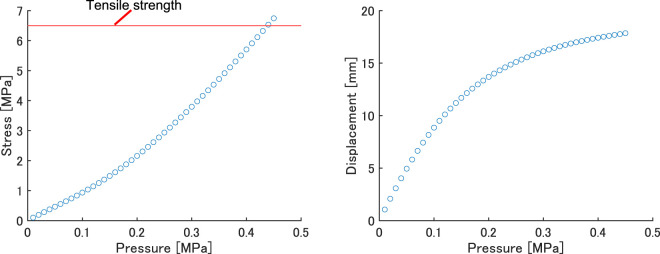
Finite-element-method predictions of stress and displacement as functions of applied pressure.

A pressure of 0.4 MPa was applied to the inner wall considering the data obtained in the previous section. The behavior of the prototype robot at 0, 0.1, 0.2, 0.3, and 0.4 MPa is shown in Figure 10, and the maximum stress value at each applied pressure is shown in Figure 11. From Figure 10, it can be seen that the leg flexed toward the lower rear side when the pressure was applied. The displacement increased with increasing pressure. From Figure 11, the stress when the applied pressure was less than 0.3 MPa did not exceed the tensile strength of KE-1600, but when 0.4 MPa was applied, the stress exceeded the tensile strength, potentially causing the robot to break. Therefore, the robot should be designed so that the pressure generated by the FRT is 0.3 MPa.

### 3.2 Manufacturing process

**FIGURE 12:**
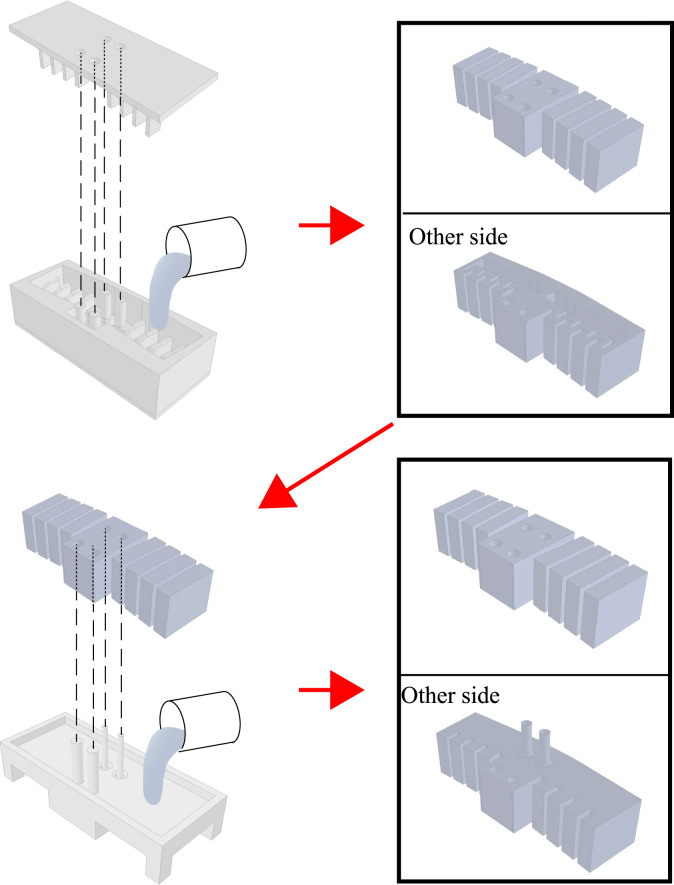
Diagram showing the manufacturing procedure. Blue = silicone, gray = mold.

The soft robot part was made by pouring KE-1600 into a mold that was divided into three elements (lower, side, and upper), with a bottom mold covering the cavity created on the lower side. Although the lower and side parts could be fabricated as a single element, they were fabricated separately for easy removal of the molded material. The mold was fabricated using a fused-deposition-modeling 3D printer. The procedure was as follows (see also Figure 12):

1. The silicone was mixed at the prescribed mass ratio and thoroughly agitated using a mixer (Shinky, ARE-310). There were two stages of mixing: 30 s at 2000 rpm and 45 s at 2,200 rpm. The mixing was repeated twice.
2. The silicone was placed in a vacuum chamber for de-aeration and then poured into the lower and middle portions of the mold about three times, also for de-aeration.
3. Once the mold was filled with silicone, the upper mold was fitted and clamped to it with no gaps.
4. The silicone was cured at room temperature for about 24 h, then removed from the mold. After that, fresh silicone was poured into the bottom part of the mold in the same manner as described in 1–2 above, and the soft robot removed from the mold was attached with the bottom part of the robot facing down.
5. After the robot was cured at room temperature for about 24 h, the pressure supply tube was bonded to the air intake and the FRT to the FRT connection port using instant adhesive (Henkel, Loctite 401).

### 3.3 Experimental evaluation

**FIGURE 13:**
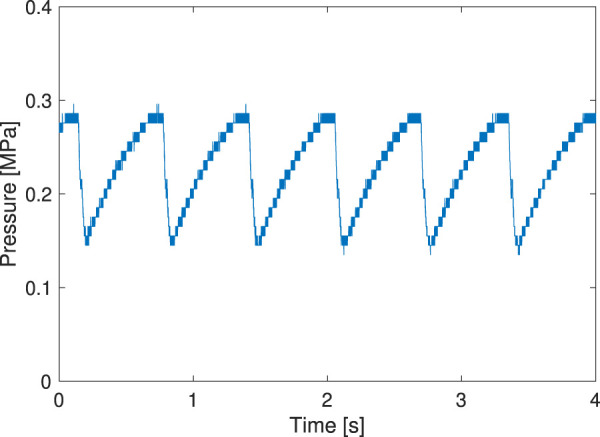
Pressure profile of the prototype driving in the air.

**FIGURE 14:**
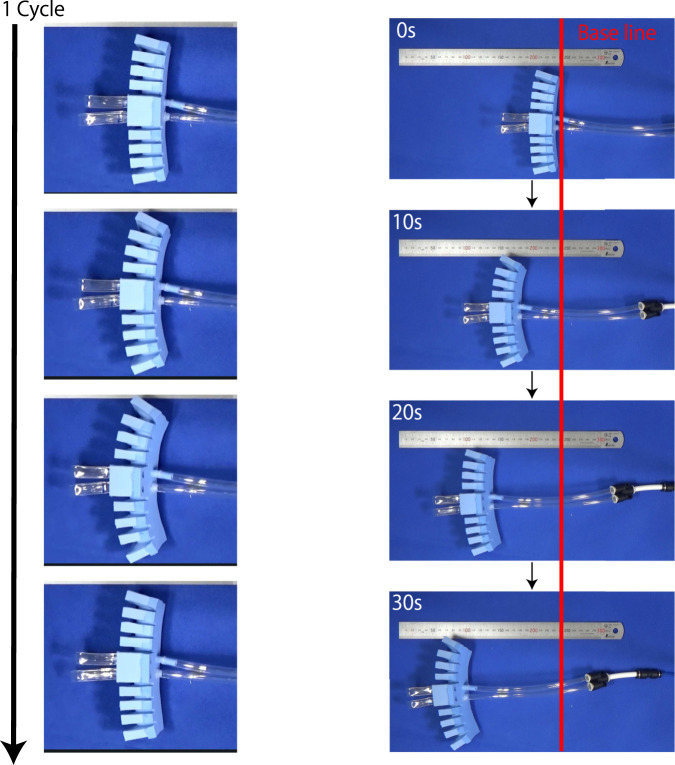
Robot walking experiment. The left figure shows four states of the robot in one cycle.

Before conducting the driving experiment on the ground, we show a pressure profile of the prototype driving in the air, as shown in Figure 13. We confirmed that the valve successfully drove the prototype robot it was installed on. The walking experiment is shown in Figure 14. Comparing the robot’s position at 0 s with that at 20 s, we can see that the robot moved 104 mm. This indicates that it was moving at a speed of 5.2 mm/s. The FRT oscillated at 1.5 Hz; thus, the robot moved 3.5 mm in one oscillation period.

## 4 Conclusion

In this study, an FRT was employed in a self-excited pneumatic valve with a simple structure. First, the pressure in the valve prototype was measured. The experiment showed the effects of length and flow rate on the frequency and amplitude of the self-excited pressure oscillations. We then manufactured a prototype locomotive robot using the pneumatic valve. The prototype could walk with two legs; it traveled 104 mm in 20 s with 1.5 Hz oscillation frequency of the valve. The results demonstrated the applicability of the valve of the proposed method fundamentally. Although self-excited actuation is particularly useful for small robots and multi-degree-of-freedom robotic systems, many challenges remain. Miniaturization and easy mass manufacturing of the device are necessary for its application to robotic systems. Because of size constraints, the valve must be directly connected to the air tank in some cases. Hence, a design method that takes into account the effect of the tank volume on the operating performance is required. In addition, it is important to develop a design method for the cooperative operation of the multiple self-excited valves. Future research should also address the interesting possibility of the valve adaptively changing its driving characteristics depending on the environment with which it is in contact. The reason is the mechanical interaction between the FRT and its surroundings.

## Data Availability

The original contributions presented in the study are included in the article/Supplementary Material, further inquiries can be directed to the corresponding author.
